# Variational Principles for Buckling of Microtubules Modeled as Nonlocal Orthotropic Shells

**DOI:** 10.1155/2014/591532

**Published:** 2014-08-05

**Authors:** Sarp Adali

**Affiliations:** Discipline of Mechanical Engineering, University of KwaZulu-Natal, Durban 4041, South Africa

## Abstract

A variational principle for microtubules subject to a buckling load is derived by semi-inverse method. The microtubule is modeled as an orthotropic shell with the constitutive equations based on nonlocal elastic theory and the effect of filament network taken into account as an elastic surrounding. Microtubules can carry large compressive forces by virtue of the mechanical coupling between the microtubules and the surrounding elastic filament network. The equations governing the buckling of the microtubule are given by a system of three partial differential equations. The problem studied in the present work involves the derivation of the variational formulation for microtubule buckling. The Rayleigh quotient for the buckling load as well as the natural and geometric boundary conditions of the problem is obtained from this variational formulation. It is observed that the boundary conditions are coupled as a result of nonlocal formulation. It is noted that the analytic solution of the buckling problem for microtubules is usually a difficult task. The variational formulation of the problem provides the basis for a number of approximate and numerical methods of solutions and furthermore variational principles can provide physical insight into the problem.

## 1. Introduction

Understanding the buckling characteristics of microtubules is of practical and theoretical importance since they perform a number of essential functions in living cells as discussed in [1–4]. In particular, they are the stiffest components of cytoskeleton and are instrumental in maintaining the shape of cells [1, 5]. This is basically due to the fact that microtubules are able to support relatively large compressive loads as a result of coupling to the surrounding matrix. Since this function is of importance for cell mechanics and transmission of forces, the study of the buckling behavior of microtubules provides useful information on their biological functions. Consequently the buckling of microtubules has been studied employing increasingly more complicated continuum models which are often used to simulate their mechanical behavior and provide an effective tool to determine their load carrying capacity under compressive loads. The present study facilitates this investigation of the microtubule buckling problem by providing a variational setting which is the basis of a number of numerical and approximate solution methods.

Buckling of microtubules occurs for a number of reasons such as cell contraction or constrained microtubule polymerization at the cell periphery. To better understand this phenomenon, an effective approach is to use continuum models to represent a microtubule. These models include Euler-Bernoulli beam by Civalek and Demir [6], Timoshenko beam by Shi et al. [7], and cylindrical shells by Wang et al. [8] and Gu et al. [9]. The present study provides a variational formulation of the buckling of microtubules using an orthotropic shell model to represent their mechanical behavior. Variational principles form the basis of a number of computational and approximate methods of solution such as finite elements, Rayleigh-Ritz and Kantorovich. In particular Rayleigh quotient provides a useful expression to approximate the buckling load directly. As such the results presented can be used to obtain the approximate solutions for the buckling of microtubules as well as the variationally correct boundary conditions which are derived using the variational formulation of the problem.

Continuum modeling approach has been used effectively in other branches of biology and medicine [10], and their accuracy can be improved by implementing nonlocal constitutive relations for micro- and nanoscale phenomenon instead of classical local ones which relate the stress at a given point to the strain at the same point. As such local theories are of limited accuracy at the micro- and nanoscale since they neglect the small scale effects which can be substantial due to the atomic scale of the phenomenon. Recent examples of microtubule models based on the local elastic theory include [11–18] where Euler-Bernoulli and higher order shear deformable beams and cylindrical shells represented the microtubules. A review of the mechanical modeling of microtubules was given by Hawkins et al. [19] and a perspective on cell biomechanics by Ji and Bao [20].

In the present study the formulation is based on the nonlocal theory which accounts for the small scale effects and improves the accuracy. The nonlocal theory was developed in the early seventies by Eringen [21, 22] and recently applied to micro- and nanoscale structures. Nonlocal continuum models have been used in a number of studies to investigate the bending and vibration behavior of microtubules using nonlocal Euler-Bernoulli [6, 23] and Timoshenko beams [24]. There have been few studies on the buckling of microtubules based on a nonlocal theory. Nonlocal Timoshenko beam model was employed in [25–27] and nonlocal shell model in [1]. In a series of studies, Shen [28–30] used nonlocal shear deformable shell theory to study the buckling and postbuckling behavior of microtubules. Nonlocal problems also arise in other subject areas and have been studied using fractional calculus in a number studies [31, 32]. The models based on beam or isotropic shell theories neglect the directional dependence of the microtubule properties. The accuracy of a continuum model can be improved further by employing an orthotropic shell theory to take this directional dependence into account as discussed in [12, 33–35].

The objective of the present study is to derive a variational principle and Rayleigh quotient for the buckling load as well as the applicable natural and geometric boundary conditions for a microtubule subject to a compressive load. The particular model used in the study is a nonlocal orthotropic shell under a compressive load with the effect of filament network taken into account as an elastic surrounding. Moreover the pressure force on the microtubule exerted by the viscous cytosol is calculated using the Stokes flow theory [1]. The inclusion of these effects in the governing equations is important to model the phenomenon accurately since it is known that the microtubules can carry large compressive forces by virtue of the mechanical coupling between the microtubules and the surrounding elastic filament network as observed by Brangwynne et al. [36] and Das et al. [37]. Moreover, the microtubules are surrounded by the viscous cytosol in addition to the soft elastic filament network and the buckling causes the viscous flow of the cytosol [38]. These two processes result in an external stress field which improves the buckling characteristics of microtubules.

Variational formulations were employed in a number of studies involving microtubules. In particular, small scale formulations for the linear vibrations of microtubules were derived using the energy expression in [39, 40] and for nonlinear vibrations in [41]. Previous studies on variational principles involving nanoscale structures include multiwalled carbon nanotubes. In particular, variational principles were derived for nanotubes under buckling loads [42], for nanotubes undergoing linear vibrations [43], and for nanotubes undergoing nonlinear vibrations [44] using the nonlocal Euler-Bernoulli beam theories. Variational principles were also derived for nanotubes undergoing transverse vibrations using a nonlocal Timoshenko beam model in [45] and a strain-gradient cylindrical shell model in [46]. Apart from providing an insight into a physical problem, the variational formulations are often employed in the approximate and numerical solutions of the problems, in particular, in the presence of complicated boundary conditions [47]. Moreover natural boundary conditions can be easily derived from the variational formulation of the problem. In the present study variational formulation of a problem is derived by the semi-inverse method developed by He [48–51] which was applied to several problems of mathematical physics to obtain the variational formulations for problems formulated in terms of differential equations [52–55]. Recently the semi-inverse method was applied to the heat conduction equation in [56] to obtain a constrained variational principle. The equivalence of this formulation to the one obtained by He and Lee [57] has been shown in [58, 59]. A recent application of the semi-inverse method involves the derivation of variational principles for partial differential equations modeling water transport in porous media [60].

## 2. Equations Based on Nonlocal Elastic Theory

**Figure 1:**
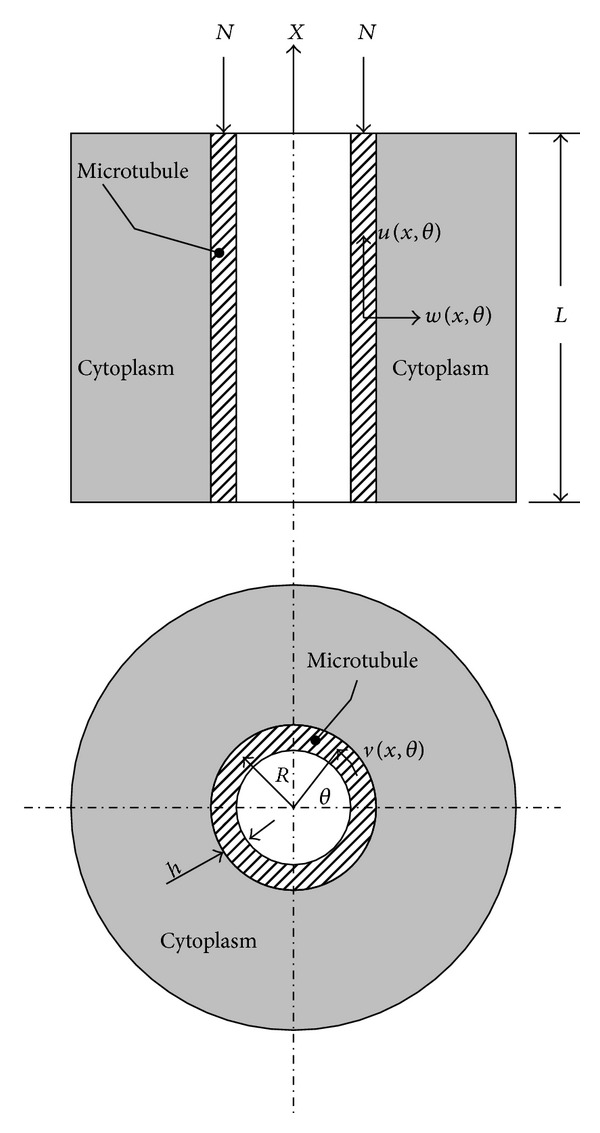
Microtubule under a compressive load and its surrounding.

In the present study the microtubule is modeled as an orthotropic cylindrical shell of length *L*, radius *R*, and wall thickness *h* and surrounded by a viscoelastic medium (cytoplasm). It is subject to an axial compressive load *N* as shown in Figure 1.

The microtubule has the Young's moduli *E*_1_ and *E*_2_ along the axial and circumferential directions, shear modulus *G* and Poisson's ratios *μ*_1_ and *μ*_2_ along the circumferential and axial directions. Dimensionless longitudinal direction is denoted by *x* = *X*/*R* and the circumferential direction by *θ* as in Figure 1. For an orthotropic shell, the constitutive relations based on nonlocal elasticity theory are given by (see [1])

(1) σx−ea02R2∇2σx=E11−μ1μ2εx+E2μ21−μ1μ2εθ,σθ−ea02R2∇2σθ=E21−μ1μ2εθ+E1μ11−μ1μ2εx,τxθ−ea02R2∇2τxθ=Gεxθ,

where ∇^2^ = (∂^2^/∂*x* ^2^)+(∂^2^/∂*θ* ^2^) is the Laplace operator, *σ*_*x*_, *σ*_*θ*_, and *τ*_*xθ*_ are stress components, and *ε*_*x*_, *ε*_*θ*_, and *ε*_*xθ*_ are the normal and shear strains. In ((1)), *ea*_0_ is the small scale parameter reflecting the nanoscale of the phenomenon and has to be experimentally determined. The differential equations governing the buckling of the microtubules are given in [1] based on the nonlocal constitutive relation ((1)). The differential equation formulation of the problem is expressed as a system of partial differential equations given by

(2) D1u,v,wL1u+M1v,w=0,D2u,v,wL2v+M2u,w=0,D3u,v,wL3w+M3u,v=0,

where *u*(*x*, *θ*), *v*(*x*, *θ*), and *w*(*x*, *θ*) are the displacement components in the axial, circumferential, and radial directions, respectively, as shown in Figure 1. The differential operators *L*_*i*_ and *M*_*i*_ are defined as

(3) $$\begin{array}{c} L_{1}\left(u\right)=k_{2}\left(1+c^{2}\right)u_{\theta \theta }+u_{xx}+\frac{N}{K}L\left(u_{xx}\right), \end{array}$$

(4) $$\begin{array}{c} M_{1}\left(v,w\right)=\left(k_{2}+\mu_{1}\right)v_{x\theta }+\mu_{1}w_{x}-c^{2}w_{xxx}+c^{2}k_{2}w_{x\theta \theta }, \end{array}$$

(5) $$\begin{array}{c} L_{2}\left(v\right)=k_{2}\left(1+3c^{2}\right)v_{xx}+k_{1}v_{\theta \theta }+\frac{N}{K}L\left(v_{xx}\right), \end{array}$$

(6) $$\begin{array}{c} M_{2}\left(u,w\right)=\left(k_{2}+\mu_{1}\right)u_{x\theta }+k_{1}w_{\theta }-c^{2}\left(3k_{2}+\mu_{1}\right)w_{xx\theta }, \end{array}$$

(7) L3w=−1+c2k1w−2c2k1wθθ −c2wxxxx+k1wθθθθ+4k2+2μ1wxxθθ +ςRKLw+NKLwxx+RKLPYY,

(8) M3u,v=−μ1ux+c2uxxx−k2uxθθ −k1vθ+c23k2+μ1vxxθ,

where the differential operator *L*(·) is defined as *L*(·) = *η* ^2^∇^2^ − 1, the subscripts *x*, *θ* denote differentiation with respect to that variable, and the dimensionless small scale parameter *η* is given by *η* = *ea*_0_/*R*. The symbols in ((3))–((8)) are defined as

(9) k1=E1E2,k2=G1−μ1μ2E1,c2=h0312R2h,K=E1h1−μ1μ2,

where *h*_0_ is the effective thickness for bending. The symbol *ς* appearing in ((7)) is the elastic constraint from the filaments network and is given by *ς* = 2.7 *E*_*c*_ where *E*_*c*_ is the elastic modulus of the surrounding viscoelastic medium. The radial pressure *P*_*YY*_ exerted by the motion of cytosol in ((7)) is computed from the dynamic equations of cytosol given in [1].

## 3. Variational Formulation

Following the semi-inverse method, we construct a variational trial-functional *V*(*u*, *v*, *w*) as follows:

(10) Vu,v,wV1u+V2v+V3w+∫02π∫0lFu,v,wdx dθ,

where *l* = *L*/*R*, *x* ∈ [0, *L*/*R*] and the functionals *V*_1_(*u*), *V*_2_(*v*), *V*_3_(*w*) are given by

(11) V1u=12∫02π∫0l−k21+c2uθ2−ux2+NKη2uxx2+uxθ2+ux2dx dθ,V2v=12∫02π∫0l−k21+3c2vx2−k1vθ2+NKη2vxx2+vxθ2+vx2dx dθ,V3w=12∫02π∫0l−1+c2k1w2+2c2k1wθ2−c2wxx2+k1wθθ2−4k2+2μ1wxθ2dx dθ⋯ +12∫02π∫0l−ςRKη2wx2+wθ2+w2+NK×η2wxx2+wxθ2+wx2+2RKLPYYwdx dθ.

In ((10)), *F*(*u*, *v*, *w*) is an unknown function to be determined such that the Euler-Lagrange equations of the variational functional ((10)) correspond to the differential equation ((2)). This establishes the direct relation between the variational formulation and the governing equations in the sense that differential equation ((2)) can be obtained from the derived variational principle using the Euler-Lagrange equations. It is noted that the choice of the trial functionals defined by ((10))-((11)) is not unique. The review article by He [61] provides a systematic treatment on the use of semi-inverse method for the derivation of variational principles and the selection of trial functionals as well as on variational methods for the solution of linear and nonlinear problems.

It is noted that the Euler-Lagrange equations of the variational functional *V*(*u*, *v*, *w*) are

(12) L1u+δFδu=0,L2v+δFδv=0,L3w+δFδw=0.

Thus the functionals *V*_1_(*u*), *V*_2_(*v*), and *V*_3_(*w*) are the variational functionals for the differential operators *L*_1_(*u*), *L*_2_(*v*), and *L*_3_(*w*), respectively. In ((12)), the variational derivative *δF*/*δu* is defined as

(13) δFδu∂F∂u−∂∂x∂F∂ux−∂∂θ∂F∂uθ+∂2∂x2∂F∂uxx+∂2∂x∂θ∂F∂uxθ+∂2∂θ2∂F∂uθθ−∂3∂x3∂F∂uxxx⋯.

Comparing ((12)) with ((2)), we observe that the following equations have to be satisfied for Euler-Lagrange equations of *V*(*u*, *v*, *w*) to represent the governing equation ((2)), namely,

(14) δFδu=M1v,w=k2+μ1vxθ+μ1wx −c2wxxx+c2k2wxθθ,

(15) δFδv=M2u,w=k2+μ1uxθ+k1wθ −c23k2+μ1wxxθ,

(16) δFδw=M3u,v=−μ1ux+c2uxxx−k2uxθθ −k1vθ+c23k2+μ1vxxθ.

The function *F*(*u*, *v*, *w*) has to be determined such that ((14))–((16)) are satisfied. For this purpose we first determine *F*(*u*, *v*, *w*) satisfying ((14)) to obtain

(17) Fu,v,w−k2+μ1vxuθ+μ1wxu+c2wxxux+c2k2wxuθθ+Φv,w,

where Φ(*v*, *w*) is an unknown function of *v* and *w*. Next we compute *δF*/*δv* from ((17)), namely,

(18) $$\begin{array}{c} \frac{\delta F}{\delta v}=\left(k_{2}+\mu_{1}\right)u_{x\theta }+\frac{\delta \Phi \left(v,w\right)}{\delta v} \end{array}$$

which should satisfy ((15)). From ((15)) and ((18)) it follows that

(19) $$\begin{array}{c} \frac{\delta \Phi \left(v,w\right)}{\delta v}=k_{1}w_{\theta }-c^{2}\left(3k_{2}+\mu_{1}\right)w_{xx\theta }. \end{array}$$

The expression for Φ(*v*, *w*) satisfying ((19)) is determined as

(20) $$\begin{array}{c} \Phi \left(v,w\right)=-k_{1}wv_{\theta }+c^{2}\left(3k_{2}+\mu_{1}\right)w_{xx}v_{\theta }. \end{array}$$

Thus from ((17)) and ((20)), *F*(*u*, *v*, *w*) is obtained as

(21) Fu,v,w−k2+μ1vxuθ+μ1wxu+c2wxxux+c2k2wxuθθ−k1wvθ+c23k2+μ1wxxvθ.

Finally we note that ∂*F*/∂*w* satisfies ((16)). Thus the function *F*(*u*, *v*, *w*) given by ((21)) satisfies the Euler-Lagrange equations ((14))–((16)) as required. Now the variational functional can be expressed as

(22) $$\begin{array}{c} V\left(u,v,w\right)=V_{1}\left(u\right)+V_{2}\left(v\right)+V_{3}\left(w\right)+V_{4}\left(u,v,w\right), \end{array}$$

where

(23) V4u,v,w=∫02π∫0lFu,v,wdx dθ=∫02π∫0l−k2+μ1vxuθ+μ1wxu+c2wxxux+c2k2wxuθθ−k1wvθ+c23k2+μ1wxxvθdx dθ

and *V*_1_(*u*), *V*_2_(*v*), and *V*_3_(*w*) are given by ((11)). To verify the validity of the variational formulation ((22)), one has to show that the Euler-Lagrange equations of *V*(*u*, *v*, *w*) yield the governing ((2)). The fact that this is indeed the case can be shown easily.

## 4. Rayleigh Quotient

The Raleigh quotient is obtained for the buckling load *N* by noting that

(24) V1u=−R1u+NSu,V2v=−R2v+NSv,V3w=−R3w+NSw,

where

(25) R1u=12∫02π∫0lk21+c2uθ2+ux2dx dθ,R2v=12∫02π∫0lk21+3c2vx2+k1vθ2dx dθ,R3w=12∫02π∫0l1+c2k1w2−2c2k1wθ2+c2wxx2+k1wθθ2+4k2+2μ1wxθ2dx dθ⋯+12∫02π∫0lςRKη2wx2+wθ2+w2−2RKLPYYwdx dθ,Sy=12K∫02π∫0lη2yxx2+yxθ2+yx2dx dθ.

Thus from ((22)) and ((24)), it follows that

(26) Vu,v,w−R1u+R2v+R3u+V4u,v,w+NSu+Sv+Sw

and the Rayleigh quotient can be expressed as

(27) $$\begin{array}{c} N=\underset{u,v,w}{\min}\frac{R_{1}\left(u\right)+R_{2}\left(v\right)+R_{3}\left(w\right)-V_{4}\left(u,v,w\right)}{S\left(u\right)+S\left(v\right)+S\left(w\right)}. \end{array}$$

## 5. Natural and Geometric Boundary Conditions

It is noted that the displacements are equal at the end points *θ* = 0 and *θ* = 2*π*; that is,

(28) ux,0=ux,2π,vx,0=vx,2π,wx,0=wx,2πfor  x∈0,l.

The first variations of *V*(*u*, *v*, *w*) with respect to *δu*, *δv*, and *δw*, denoted by *δ*_*u*_ *V*, *δ*_*v*_ *V*, and *δ*_*w*_ *V*, respectively, can be obtained by integration by parts and using ((28)). We first obtain the variations of *V*_1_(*u*) and *V*_4_(*u*, *v*, *w*) with respect to *δu* which are given by

(29) δuV1u=∫02π∫0lL1uδu dx dθ+B1u,δu,δux,δuV4u,v,w=∫02π∫0lM1v,wδu dx dθ+B2w,δu,

where

(30) B1u,δu,δux =∫02π−ux−NKη2uxxx+uxθθ−uxδu+NKη2uxxδuxx=0x=ldθ,B2w,δu=∫02πc2wxxδux=0x=ldθ.

Similarly

(31) δvV2v∫02π∫0lL2vδv dx dθ+B3v,δv,δvx,δvV4u,v,w∫02π∫0lM2u,wδv dx dθ+B4u,δv,

where

(32) B3v,δv,δvx=∫02π−k21+3c2vx−NKη2vxxx+vxθθ−vxδv+NKη2vxxδvxx=0x=ldθ,B4u,δv=∫02π−k2+μ1uθx=0x=lδv dθ.

Finally we obtain *δ*_*w*_ *V*_3_(*w*) and *δ*_*w*_ *V*_4_(*u*, *v*, *w*), namely,

(33) δwV3w=∫02π∫0lL3wδw dx dθ+B5w,δw,δwx+B6w,δw,δwx,δwV4u,v,w=∫02π∫0lM3u,vδw dx dθ+B7u,v,δw,δwx+B7u,v,δw,δwx,

where

(34) B5w,δw,δwx =∫02πc2wxxx+4k2+2μ1wxθθδw−c2wxxδwxx=0x=ldθ,B6w,δw,δwx =∫02π−ςRKη2wx−NKη2wxxx+wxθθ−wxδw+NKη2wxxδwxx=0x=ldθ,B7u,v,δw,δwx =∫02πμ1u+c2k2uθθ−uxx−c23k2+μ1vxθδw+c23k2+μ1vθ+c2uxδwxx=0x=ldθ.

Since the first variations of the functional *V*(*u*, *v*, *w*) are zero, that is,

(35) $$\begin{array}{c} \delta_{u}V\left(u,v,w\right)=\delta_{v}V\left(u,v,w\right)=\delta_{w}V\left(u,v,w\right)=0 \end{array}$$

by the fundamental lemma of the calculus of variations, we have

(36) B1u,δu,δux+B2w,δu+B3v,δv,δvx+B4u,δv+B5w,δw,δwx+B6w,δw,δwx+B7u,v,δw,δwx=0

which yields the boundary conditions. We first note that ((36)) can be written as

(37) $$\begin{array}{c} \overset{5}{\underset{i=1}{\sum }}b_{i}=0, \end{array}$$

where

(38) b1u,w,δu,δux =∫02π−ux−NKη2uxxx+uxθθ−ux+c2wxxδu+NKη2uxxδuxx=0x=ldθ,b2u,v,w,δv,δvx=∫02π−k2+μ1uθ−k21+3c2vx−NKη2vxxx+vxθθ−vx−c23k2+μ1wxθδvx=0x=ldθ +∫02πNKη2vxxδvxx=0x=ldθ,b3w,δw =∫02πc2wxxx+4k2+2μ1wxθθ−ςRKη2wx−NKη2wxxx+wxθθ−wxδwx=0x=ldθ,b4u,v,δw =∫02πμ1u+c2k2uθθ−uxx−c23k2+μ1vxθδwx=0x=ldθ,b5u,v,w,δwx =∫02π−c2wxx+NKη2wxx+c23k2+μ1vθ+c2uxδwxx=0x=ldθ.

From ((37))-((38)), the natural and geometric boundary conditions are obtained at *x* = 0 and *x* = *l* as

(39) ux+NKη2uxxx+uxθθ−ux−c2wxx=0  or  u=0,uxx=0  or  ux=0,k2+μ1uθ+k21+3c2vx+NKη2vxxx+vxθθ−vx+c23k2+μ1wxθ=0  or  v=0,vxx=0  or  vx=0,c2wxxx+4k2+2μ1wxθθ−ςRKη2wx−NKη2wxxx+wxθθ−wx⋯+μ1u+c2k2uθθ−uxx−c23k2+μ1vxθ=0  or  w=0,−c2wxx+NKη2wxx+c23k2+μ1vθ+c2ux=0  or  wx=0.

## 6. Conclusions

The variational formulation for the buckling of a microtubule was given using a nonlocal continuum theory whereby the microtubule was modeled as an orthotropic shell. The continuum model of the microtubule takes the effects of the surrounding filament network and the viscous cytosol into account as well as its orthotropic properties. Methods of calculus of variations were employed in the derivation of the variational formulation and in particular the semi-inverse approach was used to identify suitable variational integrals. The buckling load was expressed in the form of a Rayleigh quotient which confirms that small scale effects lower the buckling load as has been observed in a number of studies [1, 26, 28]. The natural and geometric boundary conditions were derived using the formulations developed. The variational principles presented here form the basis of several approximate and numerical methods of solution and facilitate the implementation of complicated boundary conditions, in particular, the natural boundary conditions.
